# Microbiome-friendly PS/PVP electrospun fibrous membrane with antibiofilm properties for dental engineering

**DOI:** 10.1093/rb/rbae011

**Published:** 2024-02-09

**Authors:** Jiamin Chen, Jia Guo, Xueyun Lu, Derong Yin, Cuisong Zhou, Yuqing Li, Xuedong Zhou

**Affiliations:** State Key Laboratory of Oral Diseases, National Clinical Research Center for Oral Diseases, Department of Operative Dentistry and Endodontics, West China Hospital of Stomatology, Sichuan University, Chengdu, Sichuan, 610041, China; State Key Laboratory of Oral Diseases, National Clinical Research Center for Oral Diseases, Department of Operative Dentistry and Endodontics, West China Hospital of Stomatology, Sichuan University, Chengdu, Sichuan, 610041, China; Stomatological Hospital affiliated Suzhou Vocational Health College, Department of Operative Dentistry and Endodontics, Suzhou, 215000, China; College of Chemistry, Sichuan University, Chengdu, Sichuan, 610064, China; State Key Laboratory of Oral Diseases, National Clinical Research Center for Oral Diseases, Department of Operative Dentistry and Endodontics, West China Hospital of Stomatology, Sichuan University, Chengdu, Sichuan, 610041, China; College of Chemistry, Sichuan University, Chengdu, Sichuan, 610064, China; State Key Laboratory of Oral Diseases, National Clinical Research Center for Oral Diseases, Department of Operative Dentistry and Endodontics, West China Hospital of Stomatology, Sichuan University, Chengdu, Sichuan, 610041, China; State Key Laboratory of Oral Diseases, National Clinical Research Center for Oral Diseases, Department of Operative Dentistry and Endodontics, West China Hospital of Stomatology, Sichuan University, Chengdu, Sichuan, 610041, China

**Keywords:** *Streptococcus mutans*, PS/PVP electrospun fibrous membrane, biofilm(s), anti-adhesion, Coulomb repulsion

## Abstract

Dental caries is one of the most prevalent and biofilm-associated oral diseases in humans. *Streptococcus mutans*, with a high ability to form biofilms by adhering to hard surfaces, has been established as an important etiological agent for dental caries. Therefore, it is crucial to find a way to prevent the formation of cariogenic biofilm. Here, we report an electrospun fibrous membrane that could inhibit the adhesion and biofilm formation of *S. mutans*. Also, the polystyrene (PS)/polyvinyl pyrrolidone (PVP) electrospun fibrous membrane altered the 3D biofilm architecture and decreased water-insoluble extracellular polysaccharide production. Notably, the anti-adhesion mechanism which laid in Coulomb repulsion between the negatively charged PS/PVP electrospun fibrous membrane and *S. mutans* was detected by zeta potential. Furthermore, metagenomics sequencing analysis and CCK-8 assay indicated that PS/PVP electrospun fibrous membrane was microbiome-friendly and displayed no influence on the cell viability of human gingival epithelial cells and human oral keratinocytes. Moreover, an *in vitro* simulation experiment demonstrated that PS/PVP electrospun fibrous membrane could decrease colony-forming unit counts of *S. mutans* effectively, and PS/PVP electrospun fibrous membrane carrying calcium fluoride displayed better anti-adhesion ability than that of PS/PVP electrospun fibrous membrane alone. Collectively, this research showed that the PS/PVP electrospun fibrous membrane has potential applications in controlling and preventing dental caries.

## Graphical abstract

**Figure:**
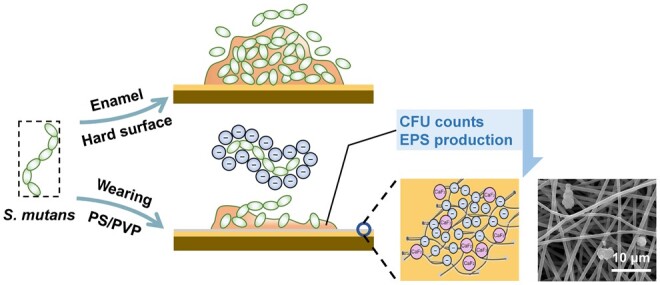

## Introduction

Dental caries is a worldwide prevalent human chronic infection that characterizes high prevalence, low treatment rate and high retreatment rate, causing severe social and economic burden. According to the reports from GBD 2016 Disease and Injury Incidence and Prevalence Collaborators, dental caries ranks in the top 10 causes of the most prevalent diseases worldwide [1]. It is a biofilm-dependent disease that is driven by the dysbiosis of oral microbiome, which can even lead to pulpitis, radical pathosis, tooth loss, maxillofacial infections or even systemic diseases [2].

*Streptococcus mutans*, the major causative agent of dental caries, maintains the stability of biofilm by expressing some virulence-related factors, including acidogenicity, aciduricity and the ability to rapidly synthesize exopolysaccharides, which initiate the tenacious adhesion of *S.mutans* to glucan-coated surfaces [3, 4]. The adhesion is mediated through sucrose-dependent and sucrose-independent mechanisms, including glucosyltransferases, glucan binding protein and the cell surface protein antigen c [5–7]. After the initial attachment of microorganisms to a surface, the formation of highly structured cell clusters, and further development and stabilization of the microcolonies [8], biofilms that are characterized by mechanical stability develop, making it difficult to eliminate only by mechanical means [9]. Therefore, inhibiting early colonization and adhesion of pathogenic microorganisms has become a key point for preventing dental caries.

Electrospinning is a handy and cost-effective technique for producing nanowebs where the fiber diameters range from a few hundred nanometers to a few microns [10]. Electrospun fibrous membranes whose physical properties can be modified by altering the structure, composition, morphology and porosity of electrospun materials under controlled conditions are characterized by high specific surface areas, porous structures and easy surface functionalization with various groups [11]. Based on the characteristics mentioned above, electrospun nanofibers have been widely studied in the biomedical field, including delivery of drugs, cell scaffolds, hemostasis, wound healing and tissue engineering [12–14].

Polystyrene (PS), which was accidentally discovered when researchers attempted to coat plastic with glass to create cultureware, has served as a basic material for culturing mammalian cells [15]. Meanwhile, uniform PS fibers can be formed using the electrospinning technique [16]. Polyvinyl pyrrolidone (PVP), synthesized by radical polymerization of the monomer N-vinypyrrolidone [17], possesses physical properties of temperature resistance, pH stability, wetting, binding, film-forming, bioactivity, non-toxicity, biodegradability and biocompatibility [18]. Furthermore, PVP is soluble in water and many organic solvents, because the polar lactam group in pyrrolidone provides hydrophilicity and non-polar methylene moiety makes PVP lipophilic [19, 20]. Because of its versatile properties, PVP is extensively used to fabricate many biomedical products via electrospinning, 3D/4D printing and other technologies [21].

PVP has many dental applications. PVP removes pigments effectively by forming complexes with compounds that cause discoloration and inhibit stain redeposition [22], so that it has been a toothpaste addictive. Also, it has an abrasive effect combined with abrasive agents and carboxymethyl cellulose [23], which is beneficial for eliminating dental plaque and maintaining toothpaste stability. PVP hydrogel and interpenetrating polymer network of poly(acrylic acid)/PVP are applied in culturing human oral mucosa cells [24, 25]. In addition, PVP-carrying drugs with low bioavailability, such as enrofloxacin and clotrimazole, can be used for drug release in the oral cavity [26, 27]. Moreover, PVP-iodine (PVP-I) oral formulations remain popular for their broad spectrum of effects, favorable tolerability profile, and additional benefits, including anti-inflammatory, anti-edematous and hemostatic effects [28]. However, the role of PS/PVP electrospun fibrous membrane in inhibiting the adhesion of microorganisms, especially *S. mutans*, is not clear.

Here, we prepared a PS/PVP electrospun fibrous membrane that inhibited the adhesion and biofilm formation of *S. mutans*. In this study, we showed that the mechanism of inhibitory effect lay in the surface potential of the electrospun fibrous membrane and *S. mutans*. Furthermore, by assessing the dynamic changes of oral microbial ecology and the cell viability of human oral keratinocytes (HOK) and human gingival epithelial cells (HGEs), it was demonstrated that PS/PVP electrospun fibrous membrane could be potentially applied to the prevention of dental caries.

## Materials and methods

### Fabrication of PS/PVP or PS fibrous membrane

The PS/PVP electrospinning membrane used in the present study was prepared according to our previously published methods [11]. Briefly, 15.8% (wt/vol) PS (Mw ∼280 000) and 9% (wt/vol) PVP (Mw ∼1,300 000) were dissolved in N,N-dimethylformamide (DMF) to prepare the electrospun solution, and the ratio of PS to PVP is about 1.75, which was used in each experiment. The mixed solution was thoroughly stirred for 24 h at room temperature. Using the electrospun apparatus, which comprised a high-voltage power supply (Series EL; Glass Coverslipsman High Voltage Inc.), a syringe pump (74900 series; Cole-Parmer Instrument Company), a syringe, a stainless-steel needle (*d* = 0.7 mm), and a grounded collector, we fabricated the PS/PVP fibrous membrane. The high voltage was set at 14 kV, made the solution surface charged, the electrostatic repulsion of which drew nanofibers. The distance between the tip of the needle and the collector was 10 cm. Then, the solution flow rate was adjusted at 0.38 ml/h by a syringe pump, and aluminum foil mounted on the collector was used to collect the PS/PVP electrospun fibers for 2 h. The ambient temperature was 25°C, with a relative humidity of 54%. Finally, the obtained PS/PVP electrospun fibrous membranes were dried at 80°C for 4 h and cut into wafers with a diameter of 1 cm.

The electrospinning fibrous membranes were dehydrated serially (30%, 40%, 50%, 60%, 70%, 80%, 85%, 90%, 95% and 100%) in ethanol and sputter-coated with gold. To calculate the average diameter, the sample was examined under a scanning electron microscope (SEM, Inspect F50; FEI, USA) at ×1000, ×5000 and ×20 000 magnifications and 100 fibers were taken into account to measure nanofiber diameter using Image J image-processing software.

Furthermore, electrospun fibrous membranes carrying calcium fluoride nanoparticles were prepared as follows. Saturated solutions of CaCl_2_ and NaF (1:1) were rapidly mixed at room temperature, and the precipitates were seen. After mixing for 16 h, the resulting aqueous suspension of CaF_2_ was centrifuged at 5000 g to collect the precipitates and the supernatant was discarded. The dried CaF_2_ was resuspended in the saturated solution CaF_2_, centrifuged at 5000 g, and dried in a vacuum. Then, CaF_2_ was resuspended in the PS/PVP electrospinning solution to prepare the modified electrospun fibrous membranes. The electrospun fibrous membranes with calcium fluoride nanoparticles were immersed in sterile deionized water, and 100 μl of the solution was collected every 24 h to detect the concentration of fluoride ions using ion chromatography (Eluent ICS-90, England), which lasted for 40 days.

### Bacterial strains and growth conditions

*Streptococcus mutans* UA159 was obtained from the American Type Culture Collection and routinely grown in brain heart infusion broth (BHI; Difco, Sparks, MD, USA) and on BHI agar at 37°C in an anaerobic incubator (10% H_2_, 5% CO_2_, and 85% N_2_). For biofilm assays, *S. mutans* was cultured in BHIS (BHI supplemented with 1% sucrose).

### Planktonic growth assays

To evaluate the impact of PS/PVP electrospun fibrous membranes on the growth of *S. mutans*, the overnight culture of *S. mutans* was subcultured into fresh BHI until reaching the mid-exponential phase (OD_600_ = 0.5) and diluted (1:100) using fresh BHI broth. Grown at 37°C, these planktonic bacteria were cultured in 12-well plates (Corning, NY, USA) containing PS/PVP electrospun fibrous membranes, glass coverslips, and hydroxyapatite discs. The medium incubated with *S. mutans* UA159 only was maintained as a control. The optical density at 600 nm (OD_600_) was measured at 2, 4, 6, 8 and 10 h using a Multiskan Spectrum (Thermo, Multiskan Go, USA). Each analysis was performed in triplicate.

### Structural imaging and analysis of biofilm

SEM and confocal laser scanning microscopy (CLSM) analyses were conducted as described previously to test the impact of PS/PVP electrospun fibrous membranes on the adhesion and biofilm structure of *S. mutans* [29]. Overnight culture of *S. mutans* was subcultured into fresh BHI until it reached the mid-exponential phase (OD_600_ = 0.5) and diluted (1:100) with BHIS and inoculated anaerobically on PS/PVP electrospun fibrous membranes and hydroxyapatite discs for 24 h at 37°C.

After 24 h, 2.5% (wt/vol) glutaraldehyde solution was used to fix the biofilm for 12 h at 4°C. Then the biofilm was rinsed with sterile PBS, dehydrated serially (30%, 40%, 50%, 60%, 70%, 80%, 85%, 90%, 95% and 100%) in ethanol, and sputter-coated with gold. The sample was examined using the SEM at ×1000, ×5000 and ×20 000 magnification.

CLSM assays were conducted to investigate the adhesion of *S. mutans* on different material surfaces. *Streptococcus mutans* biofilms were inoculated on electrospinning materials and hydroxyapatite discs as described above. During inoculation, Alexa Fluor 647 dextran conjugate (Molecular Probes; Invitrogen, Carlsbad, CA, USA) was mixed with BHIS at a final concentration of 1 μM. After incubation, the materials were transferred to another 12-well plate, washed with deionized water twice, and stained with 2.5 μM SYTO 9 (Molecular Probes; Invitrogen, Carlsbad, CA, USA) for 15 min. Images were captured with a Nikon confocal laser scanning microscope (CLSM; Nikon, N-SIM) with a ×60 oil immersion objective lens. The image collection gates were set at 495–515 nm for SYTO 9 and 655–690 nm for Alexa 647, and each biofilm was scanned at five randomly selected positions.

### Biofilm quantification

To evaluate the impact of PS/PVP electrospun fibrous membranes on the characteristic of *S. mutans* biofilms, overnight culture of *S. mutans* was subcultured into fresh BHI until it reached the mid-exponential phase (OD_600_ = 0.5), followed by diluting (1:100) with BHIS and inoculating anaerobically on PS/PVP electrospun fibrous membranes and hydroxyapatite discs for 24 h at 37°C.

For colony-forming unit (CFU) counts [30], the biofilms were scraped from the surfaces using sterile PBS and transferred into a 1.5-ml sterile centrifuge tube. Then the bacterial suspension was vortexed, serially diluted in PBS and plated onto BHI agar plates. These plates were incubated anaerobically at 37°C for 48 h to assess the microorganism viability.

The assays were performed as described previously with slight modifications for biofilm formation [31]. Briefly, the culture medium was gently decanted. The biofilms were rinsed with sterile PBS three times, fixed with 4% paraformaldehyde for 15 min and stained with 0.1% (wt/vol) crystal violet for 5 min. The bound dye was extracted with 33% (vol/vol) acetic acid, and the absorbance was recorded at 575 nm.

The amount of water-insoluble exocellular polysaccharides was determined using the anthrone method as previously described [31]. Briefly, the biofilms scraped from the surfaces were resuspended and centrifuged to collect the cell pellet. Washed twice with sterile PBS, the cell pellet was resuspended in 1M NaOH for 2 h at 37°C with shaking. The supernatant was obtained by centrifugation and mixing with three volumes of anthrone–sulfuric acid reagent at 95°C for 6 min. The absorbance was measured spectrophotometrically at 625 nm.

### Zeta potential

The zeta potential was measured as described previously with slight modifications [32]. Tetrabutylammonium bromide (TBAB), at a ratio of 1:20, was added to the PS/PVP solution. Then, the PS/PVP and PS/PVP+TBAB solutions were diluted (1:10) using DMF. The Malvern Zetasizer Nano ZS90 (Malvern Instruments Ltd, England) utilizes an electrophoretic light scattering technique to measure the zeta potential. All the tests were performed at room temperature. After measuring the zeta potential, the corresponding electrospun fibrous membranes were prepared as described above.

### Cell viability

To evaluate the biocompatibility of electrospun fibrous membranes, a CCK-8 assay was performed on HOKs and HGEs according to the manufacturer’s instructions (Cell Counting-Kit-8, DOJINDO, Japan) and previous protocol with slight modifications [33]. Briefly, the cells were cultured in DMEM (Gibco) supplemented with 20% FBS (Gibco), 100 U/ml of penicillin, and 100 mg/ml of streptomycin in a humidified 5% CO_2_ atmosphere at 37°C at a density of 5000 cells/well in 96-well plates. After incubation with HA and PS/PVP for 24 h, the cells were washed with sterile PBS, and 0.1 ml of CCK-8 reagent was added to each well, followed by incubation at 37°C for 3 h. The blank group contained cells only. Then the absorbance was measured at 450 nm using a spectrometer (Power Wave XS2, Bio-Tek, USA).

### Effect of electrospun fibrous membranes on oral microbiota

Approximately 10 ml of unstimulated whole saliva of each healthy subject was collected at least 1 h after food intake by expectorating into 15-ml sterile centrifuge tubes. The collected saliva was mixed and stored at −80°C before the following experiment. During the experiment, the salivary samples were diluted (1:10) using BHI broth, and the diluted saliva was added to a 12-well plate containing electrospinning materials and hydroxyapatite discs; the diluted saliva alone (without material) was maintained as blank. The samples were collected at 1, 2 and 3 days to conduct metagenomics sequencing analysis to test the impact of different materials on oral microbiota.

### Preparation of enamel samples and orthodontic retainers

Enamel samples were prepared as described previously with slight modifications [34]. Bovine incisors, free of caries and cracks, were collected. To separate the crown, each incisor was cut at the cementoenamel junction using a low-speed water-cooled diamond saw (Minitom, Struers, Copenhagen, Denmark). Enamel blocks were then embedded in epoxy resin. The surfaces of the labial enamel were polished with 800-, 1200-, 1500-, 2000- and 2400-grit carbide polishing papers under running water. Partly painted with acid-resistant nail varnish, the samples left a 3 × 4-mm^2^ window. Then the specimens were ultrasonicated with an ultrasonic cleaner (FS20; Fisher Scientific, Pittsburgh, PA, USA) for 10 min to remove the smear layer. The hardness of enamel samples was tested by a Vickers hardness tester (MMT-X7A, Matsuzawa, Japan) with a diamond indenter under a 50-gf load for 10 s. The average value was calculated after creating five indentations, and only enamel samples with microhardness between 3.3 and 4.0 Gpa were saved for the following step. The enamel specimens were stored in 0.5% thymol solution at 4°C before use and sterilized in an ethylene oxide sterilizer (Anprolene AN 74i, Andersen) before the next experiment.

Concerning the orthodontic retainer, after preparing the retainer suitable for enamel samples, we attached several metal electrodes on the retainer in advance to make the retainer a collector to collect the electrospun fibers. Besides, the retainer with electrospun fibrous membranes carrying calcium fluoride nanoparticles was prepared as aforesaid.

### Statistical analysis

All the experiments were performed at least in triplicate and reproduced three separate times. Statistical analysis was performed using SPSS (Version 21.0 for Windows; SPSS Inc.) with one-way analysis of variance to compare the means of all the groups and a two-tailed Student’s *t*-test to compare the means of the two groups. A two-tailed *P* value ˂0.05 was considered statistically significant.

## Results

### The PS/PVP electrospun fibrous membrane inhibited the adhesion and biofilm formation of *S.mutans*

As shown in Supplementary Figure S1A, the electrospun fibrous membrane prepared with PS/PVP was observed under SEM. No significant difference was seen between electrospun fibers, indicating that the obtained electrospun fibrous membrane had a uniform fiber diameter and stable spatial structure. Furthermore, the average diameter of nanofiber was 0.197 μm (Supplementary Figure S1B). The results suggested that PVP, an extra macromolecular compound added to PS, had little influence on the diameter and structure of the electrospun fibrous membrane.

**Figure 1.**
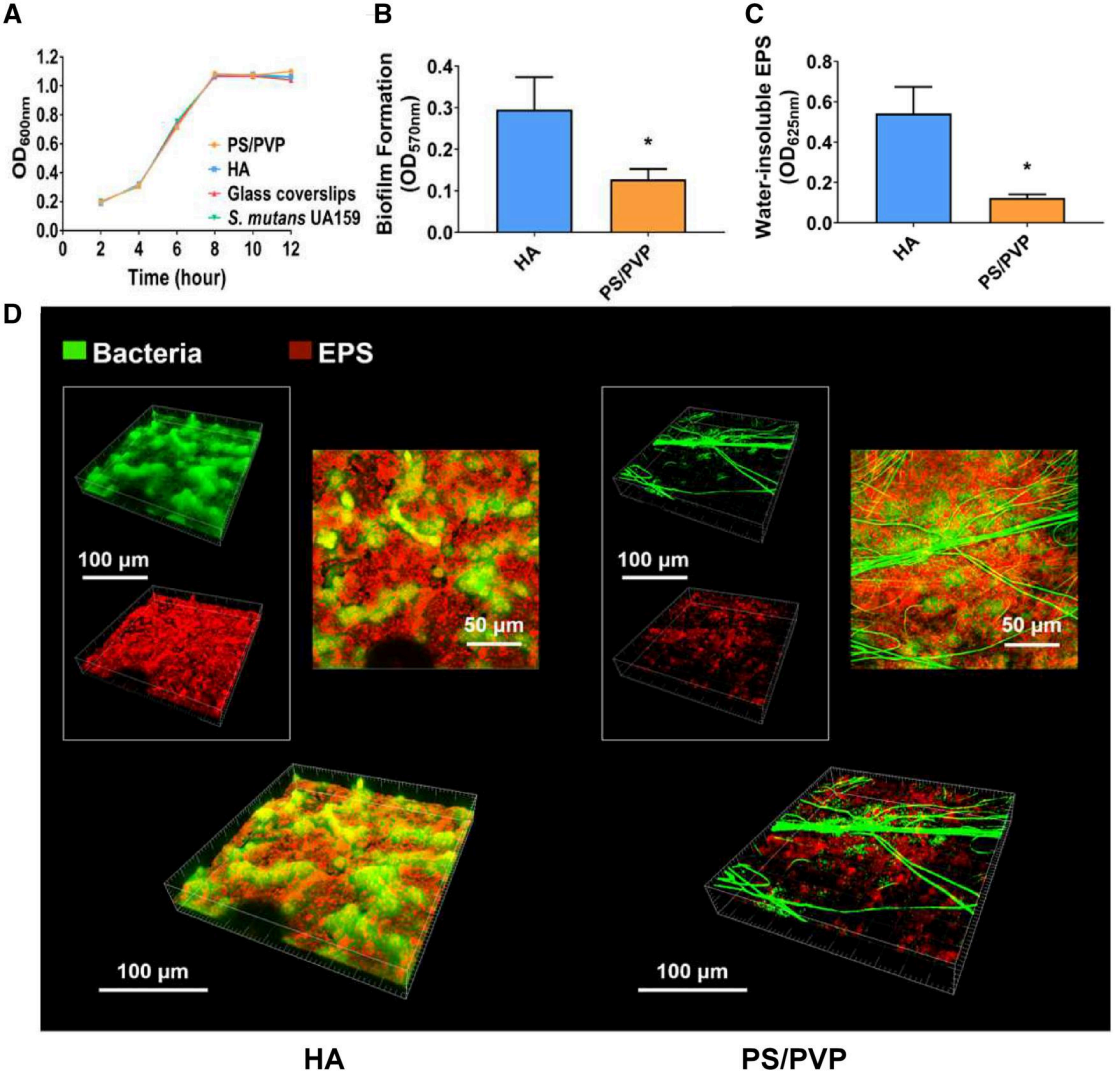
Electrospun fibrous membrane inhibits biofilm formation and adhesion of *S.mutans* UA159. (**A**) Growth curves of *S.mutans* grown in BHI medium containing electrospun fibrous membranes, glass coverslips and hydroxyapatite disc for 12 h. (**B**) Quantitative results were collected to evaluate biofilm formation on different surfaces by crystal violet staining. Data were obtained in triplicate and are presented as mean ± SD (*P ˂ 0.05). (**C**) Water-insoluble EPS content of biofilms quantified using anthrone–sulfuric method. Data were obtained in triplicate and are presented as mean ± SD (*P ˂ 0.05). (**D**) Double-labeled imaging of biofilms showing EPS (red, Alexa Fluor 647) and bacteria (green, SYTO 9). Images were taken using a ×60 oil immersion objective lens. Representative images from at least five randomly selected positions of each sample are shown.

*Streptococcus mutans* was cultured in BHI alone and BHI containing electrospun fibrous membranes, hydroxyapatite discs and glass coverslips, respectively. The growth of *S. mutans* in different materials showed similar characteristics, suggesting that the electrospun fibrous membrane did not inhibit the growth of *S. mutans* (Figure 1A). While numerous researchers have selected glass coverslips for the biofilm incubation of *S. mutans*, our study did the following experiments using hydroxyapatite discs whose chemical components were similar to enamel. Since no inhibition on the growth of *S. mutans* was observed, we wondered whether PS/PVP would affect the biofilm formation of *S. mutans*, influencing its content of water-insoluble exocellular polysaccharides. As shown in Figure 1B, biofilms grown on PS/PVP electrospun fibrous membrane showed an apparent decrease in biofilm formation than HA. The water-insoluble extracellular polysaccharide content of biofilms was quantified with the anthrone–sulfuric method. The results were similar to those of biofilm formation, i.e. a significant decrease in water-insoluble extracellular polysaccharide was detected in the PS/PVP group (Figure 1C). Furthermore, Figure 1D shows the representative images of 3D renderings of the EPS–microcolony complex with bacteria in green and EPS in red. More bacterial clusters wrapped with EPS were seen in the biofilm formed on HA. In contrast, we found that electrospun fibers were dyed in green, and fewer bacteria were surrounded with less EPS in the biofilm on PS/PVP electrospun fibrous membranes, consistent with SEM observations.

Altogether, these results suggest that PS/PVP electrospun fibrous membranes had an inhibitory effect on biofilm formation and adhesion of *S. mutans*.

### The anti-adhesion effect of PS/PVP electrospun fibrous membrane regardless of changes in the medium

**Figure 2.**
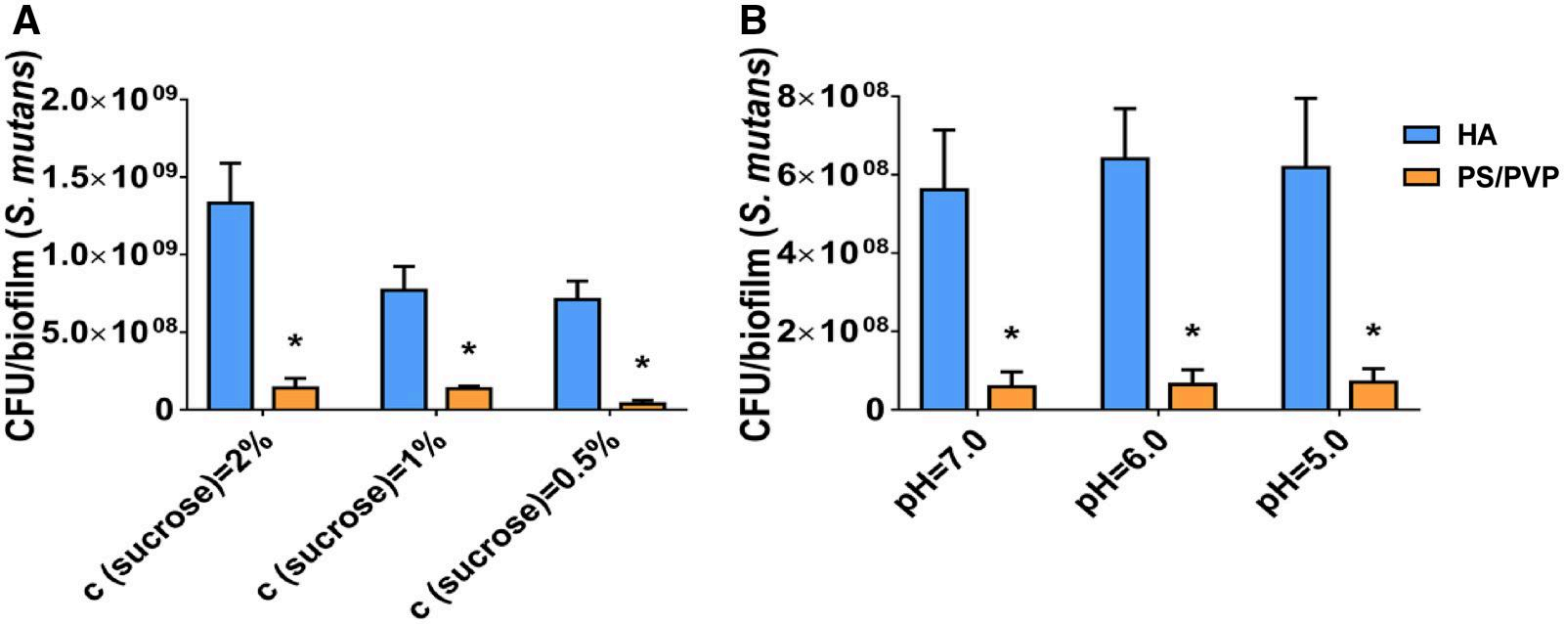
CFU counts of *S.mutans* biofilms on HA and PS/PVP electrospun fibrous membrane grown in different culture medium. CFU counts of *S.mutans* biofilms cultured at (**A**) different concentration of sucrose of medium and (**B**) different pH of medium. Data were obtained in triplicate and are presented as mean ± SD (*P ˂ 0.05).

The oral cavity is a complex environment with dynamic changes. The concentration of carbohydrates and pH will change significantly with food intake. Since we discovered that the PS/PVP electrospun fibrous membrane inhibited the adhesion of *S. mutans*, disrupting its biofilm formation, we were curious about whether the anti-adhesion effect could be altered due to the changes in the environment. Therefore, we changed the concentration of sucrose and pH of the medium to determine the impact of different environments on *S. mutans* adhesion. As shown in Figure 2A, the CFU counts of PS/PVP were significantly lower than those of HA at different concentrations of sucrose (2%, 1% and 0.5%, respectively). Also, Figure 2B shows similar findings with pH changes (7.0, 6.0 and 5.0, respectively). In other words, the electrospun fibrous membranes could inhibit the adhesion of *S. mutans* under different culture conditions.

### 3.3. The mechanism of the inhibitory effect of PS/PVP electrospun fibrous membranes on adhesion of *S.mutans*

To further determine the mechanism of PS/PVP electrospun fibrous membranes inhibiting the adhesion of *S. mutans*, we used PS/PVP and PS/PVP plates to examine whether the spatial structure played an important role. As shown in Supplementary Figure S2A, biofilms formed on HA exhibited a sieve-like structure with a large amount of extracellular polymeric substance, which formed 3D scaffolds and embedded the bacterial cells. However, the biofilms on PS/PVP plates displayed scattered and isolated EPS–microcolony complexes with less extracellular substance. On the contrary, we could hardly observe the bacterial cells and extracellular substances on PS/PVP. In addition, we compared the water-insoluble exocellular polysaccharides of the biofilms formed on HA, PS/PVP and PS/PVP plates in the mature phase (24 h) after the inoculum. The results of the production of water-insoluble exocellular polysaccharides were consistent with SEM observations; namely that the water-insoluble EPS in the PS/PVP plate was a little higher than that of PS/PVP, but both of them were distinctly less than the HA group (Supplementary Figure S2B). Besides, genes associated with exopolysaccharides synthesis and sucrose-dependent adhesion were significantly down-regulated in the PS/PVP group compared to their expression in UA159 strain (Supplementary Figure S2C). *Streptococcus mutans* synthesizes two quorum sensing (QS) signals, competence-inducing peptide and sigX-inducing peptide that are sensed through a two-component signal system and an Rgg-type intracellular transcriptional regulator, respectively [35]. Also, the genes related to QS were down-regulated in the PS/PVP group (Supplementary Figure S2D). As shown in Supplementary Figure S2E, we have observed that the hydrophilicity of PS/PVP was similar to that of PVP, while PS showed hydrophobicity. As for moderate wettable surfaces, researchers have displayed that hydrophobic surfaces showed better anti-adhesion ability for bacteria than hydrophilic surface [36]. Thus, the wettability of PS/PVP may have little influence on the anti-adhesion effect.

**Figure 3.**
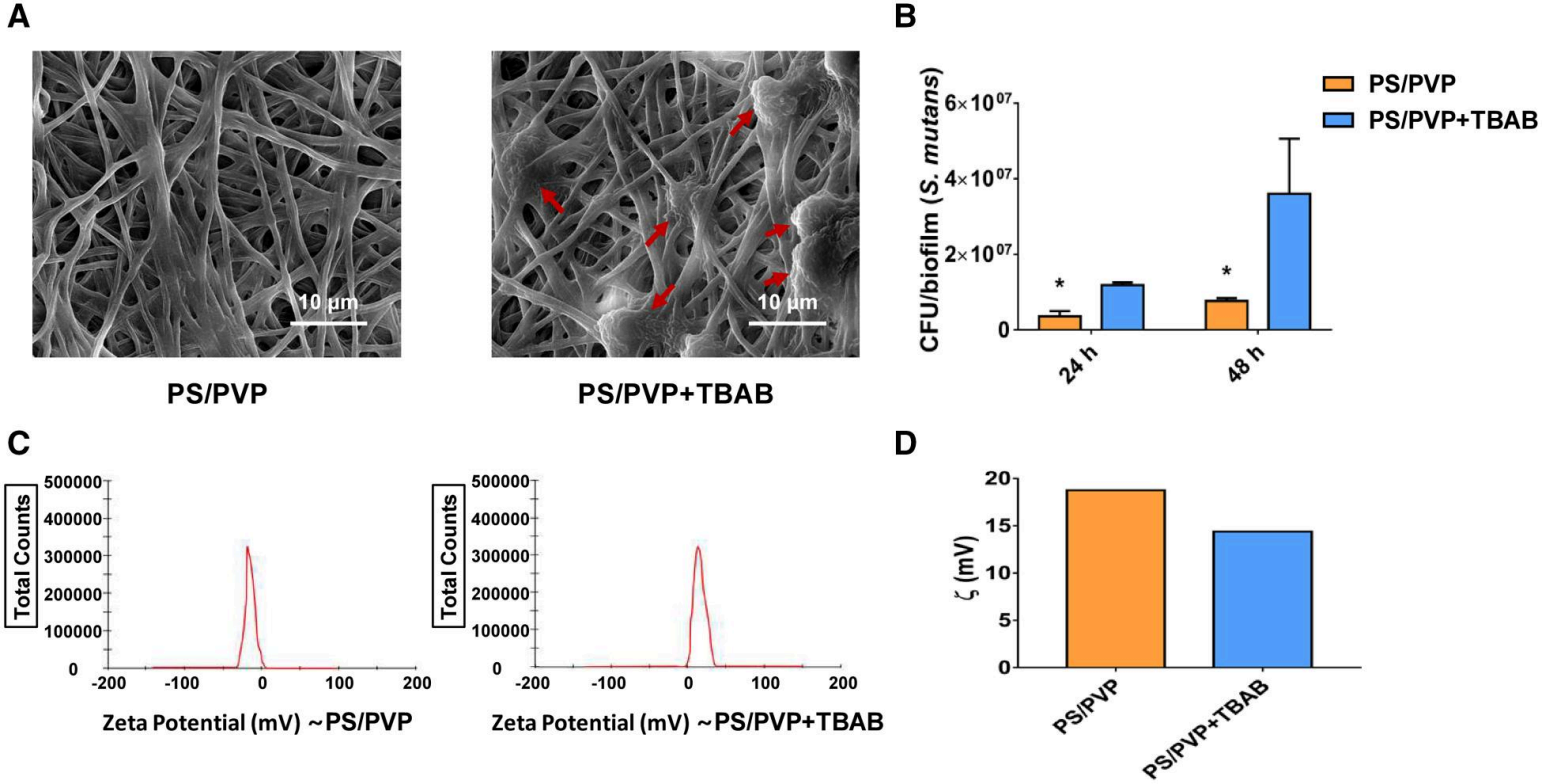
The influence of surface potential of electrospun fibrous membrane on the adhesion of *S.mutans*. (**A**) Biofilms incubated on PS/PVP and PS/PVP+TBAB, and images taken at ×5000 magnification. The red arrows indicated the bacterial cells. (**B**) CFU counts of *S.mutans* biofilms. Data were obtained in triplicate and are presented as mean ± SD (*P ˂ .05). (**C**) Zeta potential of different electrospinning solution. (**D**) Absolute zeta potential values.

In addition, we speculated whether the surface potential played a vital role in the inhibitory effect. Surprisingly, the PS/PVP+TBAB group showed apparent bacterial cells adhering to the electrospinning fibers (ESFs), whereas in the PS/PVP group, there were barely bacterial cells adhering (Figure 3A). The data on CFU counts also suggested a remarked increase in the PS/PVP+TBAB group compared to the PS/PVP group at 24 and 48 h, respectively (Figure 3B). Consequently, we examined the zeta potential of the electrospinning solution. As shown in Figure 3C, the PS/PVP solution was negatively charged. However, the PS/PVP solution processed by the surfactant, TBAB, had a positive potential. The absolute value of the zeta potential of the PS/PVP solution was higher than that of PS/PVP+TBAB (Figure 3D).

Collectively, our data indicated that it was mainly the surface potential that inhibited *S. mutans* adhesion.

### The effect of PS/PVP electrospun membrane on oral microbiota composition and cell proliferation

**Figure 4.**
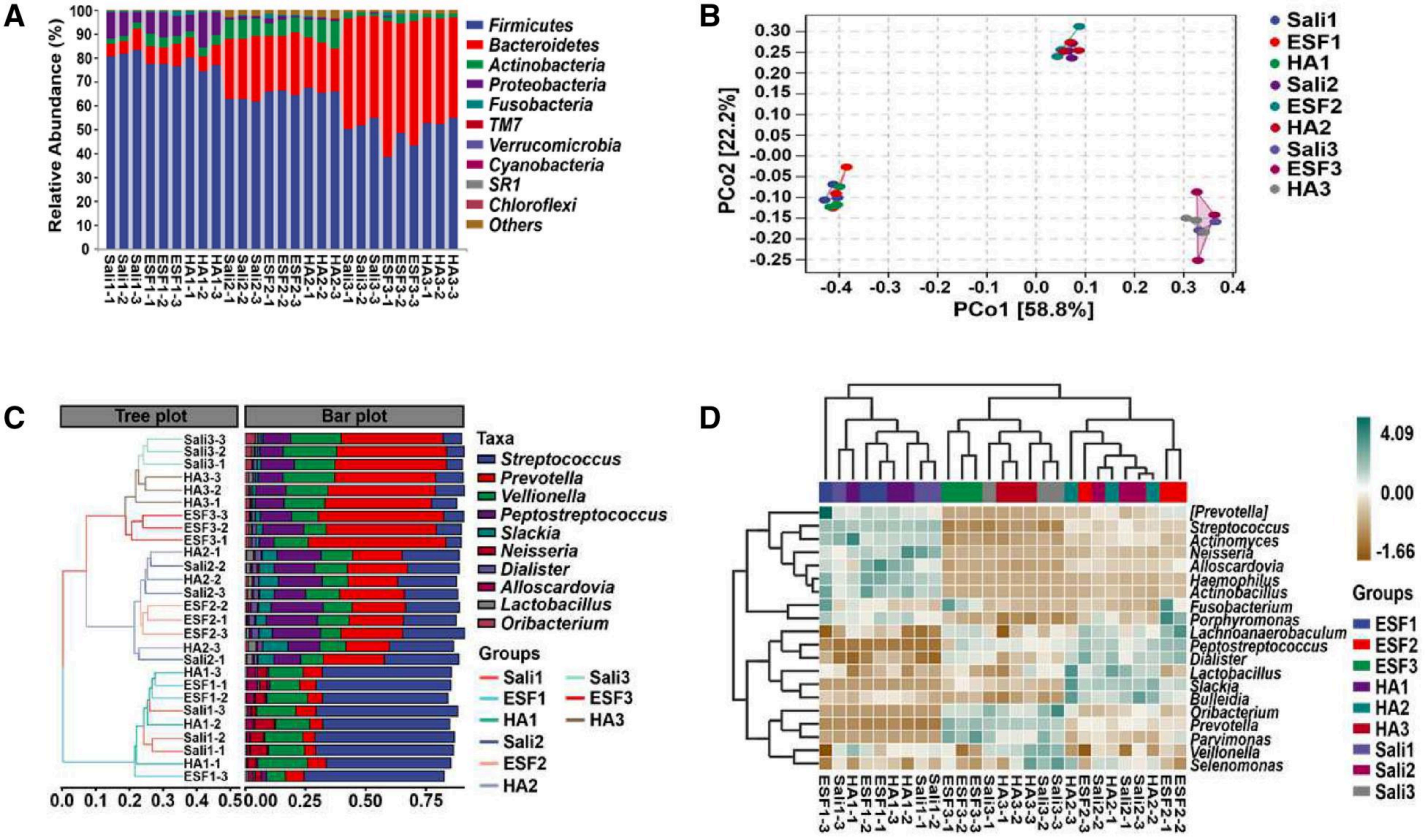
The impact of PS/PVP electrospun fibrous membrane on oral microbiota composition. (**A**) Relative abundance of oral bacteria at phylum level. (**B**) Distance matrix and principal coordinates analysis (PCoA). (**C**) Hierarchical clustering analysis. (**D**) The heatmap consisted of relative abundance of the 20 most predominant oral bacteria at genus level. ESF, electrospinning fiber; HA, hydroxyapatite disc; Sali, saliva.

Metagenomics sequencing analysis was used to further explore the effect of electrospinning materials on the oral microbiota. In all three groups, Firmicutes, Bacteroidetes, Actinobacteria and Proteobacteria comprised the vast majority of oral microbiota, and the relative abundance of Firmicutes was the highest. As the time during which the saliva was processed with ESF and HA was prolonged, the relative abundance of Bacteroidetes gradually increased, and that of Firmicutes decreased (Figure 4A). Also, there were three separate and independent areas in which various colored spots were centralized, corresponding to different processing times, indicating little difference between the experimental and healthy saliva groups at the same treatment time (Figure 4B).

Meanwhile, the data were also used for hierarchical clustering analysis. Samples were clustered according to their similarity; that is to say, the shorter the branch length between the samples, the more similar the samples were. Hence, we detected no significant changes in composition between various treatment times. In addition, *Streptococcus*, *Prevotella*, *Veillonella* and *Neisseria* were the main components on day 1, and on days 2 and 3, the abundance of *Prevotella* and *Peptostreptococcus* increased gradually, and *Streptococcus* abundance decreased (Figure 4C). Additionally, the heatmap exhibited the distribution trends of species abundance, in which the oral microbiota mainly consisting of *Streptococcus*, *Actinomyces*, *Neisseria*, *Alloscardovia*, *Haemophilus* and *Actinobacillus* on the first day shifted to *Lachnoanaerobaculum*, *Peptostreptococcus*, *Dialister*, *Lactobacillus*, *Slackia* and *Bulleidia* on the second day and *Oribacterium*, *Prevotella*, *Parvimonas*, *Veillonella* and *Selenomonas* on the third day (Figure 4D).

**Figure 5.**
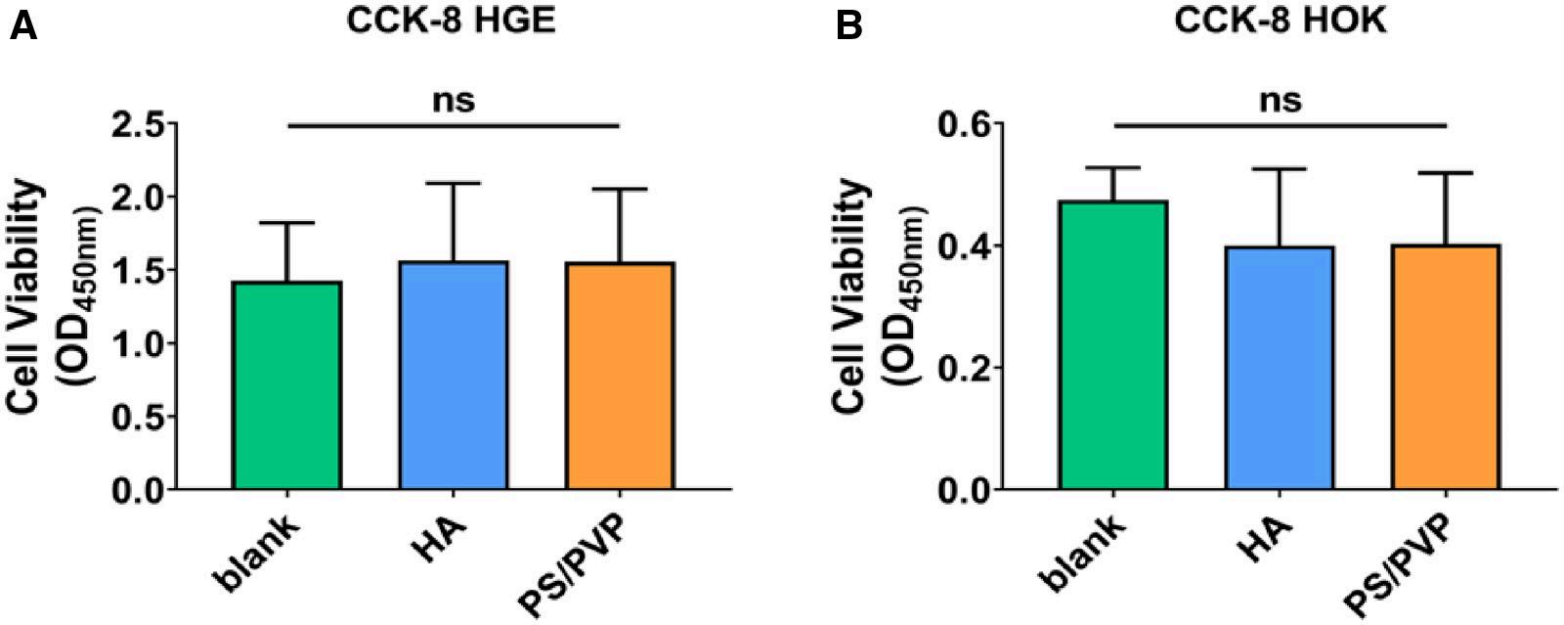
The effect of PS/PVP electrospun fibrous membrane on HGEs and HOKs. Results of CCK-8 assays performed on (**A**) HGEs and (**B**) HOKs. Data were obtained in triplicate and are presented as mean ± SD.

The CCK-8 assay was applied to analyze the proliferation of HGE and HOK in the medium containing HA and PS/PVP (Figure 5A and B). The results demonstrated no significant differences in cell viability in all the three groups.

In general, the electrospun fibrous membranes inhibited the adhesion of *S.mutans*, with no influence on oral microbial ecology and the cell viability of HGE and HOK cells.

### The PS/PVP electrospun fibrous membrane carrying calcium fluoride nanoparticles exhibited antibiofilm effect of *S.mutans*

**Figure 6.**
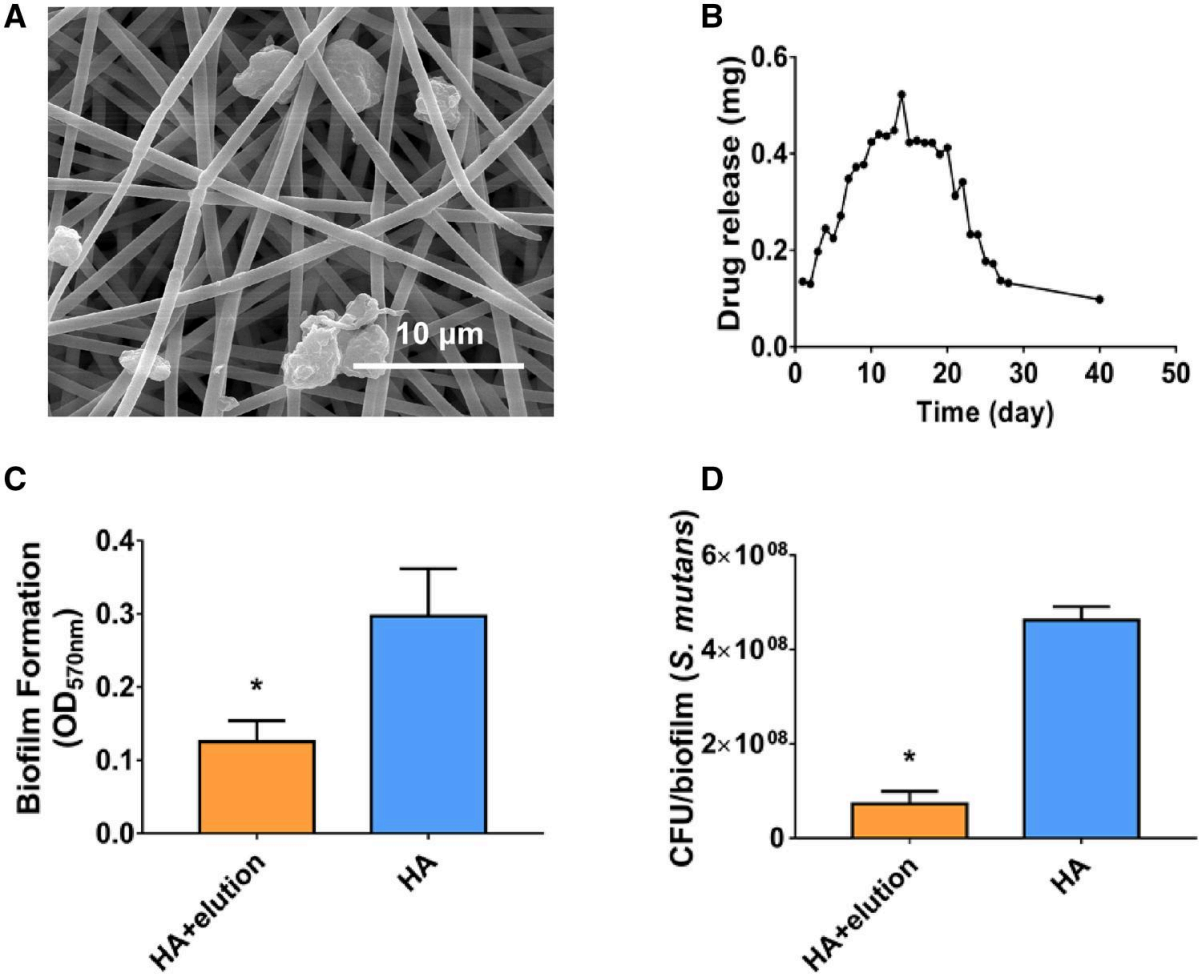
The PS/PVP electrospun fibrous membrane carrying calcium fluoride nanoparticles suppress biofilm formation of *S.mutans* UA159. (**A**) Electrospun fibrous membrane carrying calcium fluoride nanoparticles and images taken at ×10 000 magnification. (**B**) Fluoride release from modified electrospun fibrous membrane. (**C**) Data were obtained in triplicate and are presented as mean ± SD (*P ˂ 0.05). Quantitative results were collected to evaluate biofilm formation by crystal violet staining. (**D**) CFU counts of biofilms. Data were obtained in triplicate and are presented as mean ± SD (*P ˂ 0.05).

Calcium fluoride nanoparticles were carried by electrospun fibrous membranes, considering that fluoride is a clinical preventive measure to prevent dental caries. The ability of electrospun fibrous membranes to carry calcium fluoride nanoparticles was examined by SEM and determined by the elution of fluoride ions. Calcium fluoride nanoparticles were observed in electrospun fibrous membranes (Figure 6A), and Figure 6B presents the elution of soluble fluoride from calcium fluoride-doped electrospun fibrous membranes. The fluoride release first accelerated and then decelerated over the period investigated. The fluoride concentration reached the highest level at 14 days and appeared stable after 28 days. As shown in Supplementary Figure S3A, in order to test the stability and service lifetime of the films, PS/PVP electrospun membrane and PS/PVP electrospun fibrous membrane carrying calcium fluoride nanoparticles were immersed in double deionized water for 5 and 10 days. The results showed that the surface morphology and the spatial structure remain stable. However, given that after orthodontic treatment, patients need to wear retainers to maintain the therapeutic effect for 6 months or 1 year, the realistic service lifetime of PS/PVP electrospun membrane requires a longer processing time to determine. Meanwhile, tensile tests were carried out to explore the influence of the nanoparticles on the mechanical properties of the PS/PVP electrospun membrane. We observed that the tensile strength of the composite membrane was comparable to that of PS/PVP alone (Supplementary Figure S3B). Then we selected the elution at 28 days and added it to the medium to evaluate the inhibitory effect on the growth of *S. mutans*. Both biofilm formation (Figure 6C) and CFU counts (Figure 6D) exhibited a significant decrease under the influence of fluoride compared with the HA group, indicating that the concentration of fluoride at 28 days still inhibited the growth of *S. mutans*.

**Figure 7.**
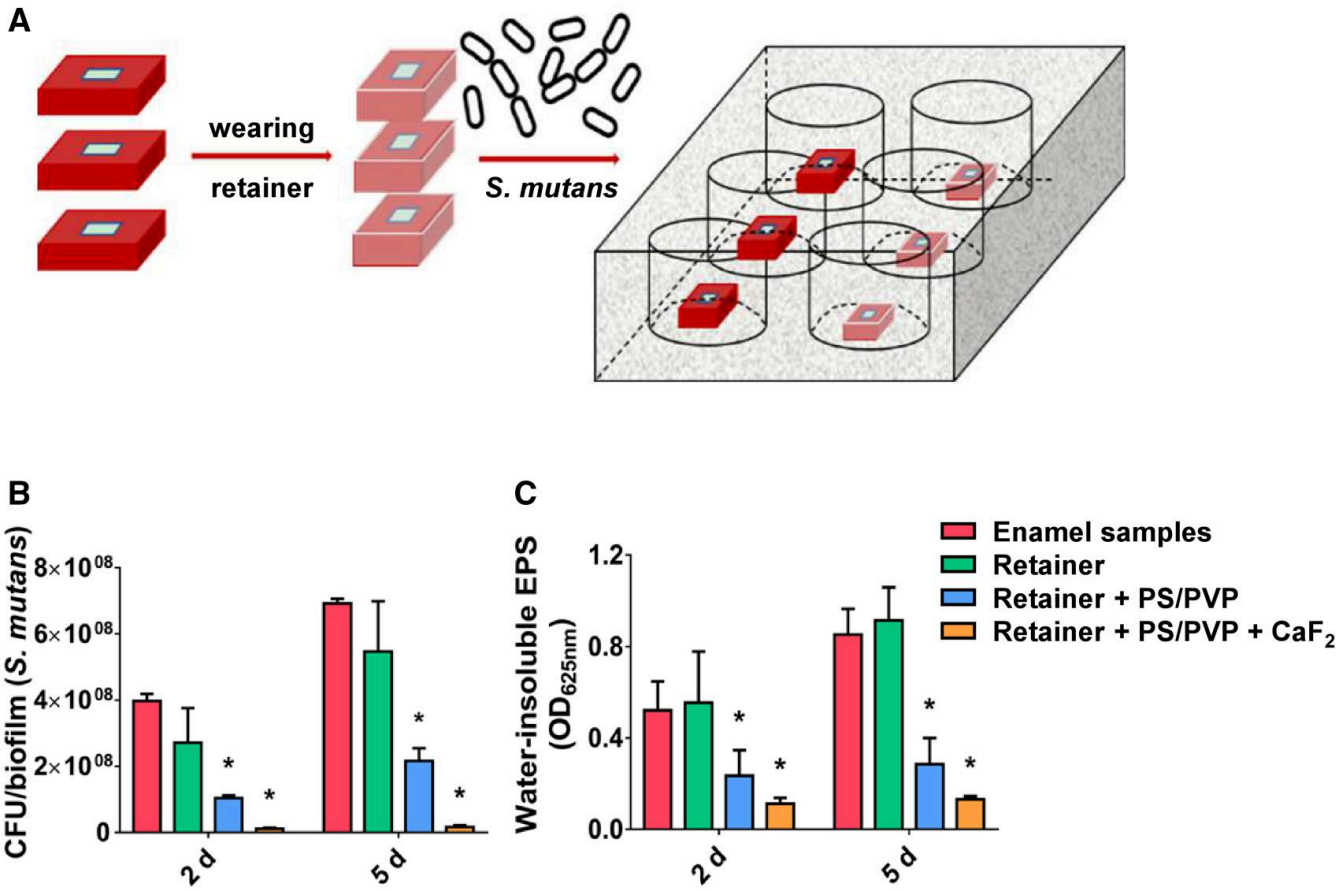
The evaluation on the antibiofilm effect of different retainers. (**A**) Schematic illustration displaying the experimental process. (**B**) CFU counts and (**C**) water-insoluble EPS content of biofilms in different groups. Data were obtained in triplicate and are presented as mean ± SD (*P ˂ 0.05).

Since the PS/PVP electrospun fibrous membranes inhibited the adhesion of *S. mutans*, with no appreciable impact on oral microbiota and cell viability, electrospun fibrous membranes may be used to fabricate retainers to prevent caries. A schematic illustration was used to show how the experimental process was performed: sterile bovine enamel samples, samples with retainers, with PS/PVP+retainer and PS/PVP+CaF_2_+retainer were placed in 6-well plates containing an *S. mutans* suspension (Figure 7A). In the *in vitro* antibiofilm experiment, we tested the CFU counts (Figure 7B) and water-insoluble exocellular polysaccharide (Figure 7C) of biofilms again on days 2 and 5, respectively. As for CFU counts, the enamel sample group exhibited the highest counts of adhered bacterial cells, followed by the retainer group, and that of the PS/PVP+retainer group was less, with the PS/PVP + CaF_2_ + retainer group exhibiting almost no bacterial cells on the surface. Concerning the water-insoluble exocellular polysaccharides, the results resembled those of CFU counts.

In a word, the retainer coated with PS/PVP electrospun fibrous membranes carrying calcium fluoride nanoparticles could effectively inhibit the adhesion of *S. mutans*, making it possible for clinical applications in orthodontic retainers.

## Discussion

Conventionally, topical fluoride regimens (such as fluoride-containing restoration) and water fluoridation for systematical use are used for caries prevention. However, the low specific surface areas of fluoride-containing restorations [37] and water fluoridation pose the potential to cause human health problems [38], which lead researchers to find new materials to prevent caries. Electrospun nanofibers used in caries prevention have been studied recently. *Garcinia mangostana* extract loaded electrospun chitosan and thiolated chitosan nanofiber could reduce oral bacteria including *S. mutans* and *Streptococcus sanguinis* in the oral cavity without cytotoxicity [39]. Sandwich-structured electrospun pH-responsive dental pastes maintain excellent hydrophobic, biocompatibility and antibacterial properties against *S. mutans* [40]. Our previous study demonstrated that bromocresol green-PS/PVP electrospun fibrous membrane could distinguish a pH of 5.5 from its adjacent pH value with higher accuracy, with potential applications in the monitoring and prevention of early caries [11]. In recent years, electrospun nanofibers have also been studied in orthodontics. During fixed orthodontic treatment, enamel demineralization around brackets is a relatively common complication, which seriously affects the aesthetics of teeth and could induce dental caries. A creative PCL (polycaprolactone)–gelatin–AgNPs (silver nanoparticles) fibers film prepared by electrospinning was made into short fibers and added to traditional orthodontic adhesives, which showed strong antibacterial properties without compromising the bonding ability [41]. In the present study, the PS/PVP electrospun fibrous membrane inhibited the adhesion of *S.mutans* and influenced the biofilm formation, suggesting that it could be a promising means as an antibiofilm/anti-caries agent. Besides, orthodontic retainers, which wear composite PS/PVP, show less adhered bacteria and water-insoluble exopolysaccharides than the retainer-only group. Our findings broaden the horizon on the application of PS/PVP electrospun fibrous membrane for orthodontic patients to prevent dental caries.

The main inorganic components of enamel and dentin are hydroxyapatite, and dental caries is mainly manifested as the demineralization and dissolution of hydroxyapatite. Under acidic conditions, fluoride can replace the hydroxyl in hydroxyapatite and transform it into fluorapatite [42]. The critical pH of fluorapatite is 4.5, which is lower than that of hydroxyapatite, thus making fluorapatite more aciduric [43]. At the same time, fluoride can accelerate the remineralization process of enamel, increasing its ability to acid resistance. Also, fluoride can inhibit the activity of enolase, which can influence glycolysis and the production of phosphoenolpyruvate in *S. mutans* [44]. Moreover, calcium fluoride is less soluble than sodium fluoride, and CaF_2_ becomes more soluble due to the formation of HF when pH decreases, maintaining the concentration of fluoride for a longer time [45, 46]. Considering the widespread application of fluoride and high specific surface areas and porous structures of electrospinning materials, PS/PVP electrospun fibrous membrane carrying calcium fluoride nanoparticles was prepared to achieve slow fluoride release for better caries prevention and increase fluoride release during microenvironment acidification. Our data further verified this speculation by measuring the elution of fluoride concentration. Besides, we used the elution of PS/PVP electrospun fibrous membrane carrying calcium fluoride nanoparticles to test the effect on biofilm formation of *S. mutans*, which showed a significant decrease.

Since a significant increase was seen in the adhered bacterial cells after the PS/PVP electrospun fibrous membrane was processed by TBAB, we speculate that the surface potential of materials influences bacterial early attachment. Considering the influence of carboxyl and acylamino of PS/PVP, the surface potential of the electrospun fibrous membrane is negative. The surface potential of *S.mutans* is also negative at pH > 3 [47]. The mechanism of negatively charged electrospinning materials repelling the negatively charged bacterial membranes could explain the results of the present study, which is called the Coulomb repulsion. TBAB is a common positively charged surfactant, forming covalent bonds with PS/PVP; therefore, the ammonium group is positively charged [48]. Studies have revealed that the positively charged surfactant, DMAHDM (Dimethylaminododecyl methacrylate), has antibacterial effects by disrupting negatively charged bacterial membranes [49]. However, there is almost no research on the antibacterial property of TBAB. The antibacterial property of TBAB and the mechanisms have not been studied thoroughly; therefore, further exploration is necessary. The results of zeta potential indicated that the surface potential was shifted to a positive charge, and the anti-adhesion ability decreased, which also explain that the anti-adhesion ability of PS/PVP plate is lower than that of PS/PVP electrospun fibrous membrane, characterized by high specific surface areas, higher surface potential, and enhanced ability to inhibit *S. mutans* adhesion.

The overgrowth of *S. mutans* in the oral biofilm, which may disrupt the oral ecological balance, is considered an important reason for the initiation and development of dental caries [50]. Therefore, targeting *S. mutans* biofilm has been suggested to be a significant therapeutic approach to prevent and control dental caries. Mechanical methods (such as brushing and flossing) are routinely used to remove the cariogenic bacteria [51]. However, these means depend on the compliance of patients to adequately control biofilm buildup [52]. What is more, patients wearing orthodontic retainers have more difficulty in maintaining oral hygiene, because retainers are hard to clean and could create spaces conducive to biofilm formation [53–55]. Intriguingly, our findings demonstrated that PS/PVP electrospun fibrous membrane not only displayed stable inhibitory effects regardless of the changes in pH and concentration of glucose of the medium but also hardly influenced the oral microbial ecology and the cell viability of HOKs and HGEs, indicating the possibility that it could be applied in the clinic to prevent dental caries.

## Conclusion

In summary, our findings suggest that the PS/PVP electrospun fibrous membrane inhibits the adhesion of *S. mutans* for that both are negatively charged which repel each other and thereby influence biofilm formation. Also, calcium fluoride-doped PS/PVP electrospun fibrous membrane can inhibit biofilm formation more effectively due to the slow release of fluoride. Furthermore, it is noteworthy that the potential clinical application of PS/PVP electrospun fibrous membrane to prevent dental caries considers the preliminary study of biocompatibility.

## Supplementary Material

rbae011_Supplementary_Data

## Data Availability

The data underlying this article are available in the article and in its online supplementary material.
