# Supplementary figures and images for: Microbiome-friendly PS/PVP electrospun fibrous membrane with antibiofilm properties for dental engineering
Source: Regen Biomater. 2024 Feb 9;11:rbae011. doi: 10.1093/rb/rbae011 (PMC10898674; doi:10.1093/rb/rbae011)

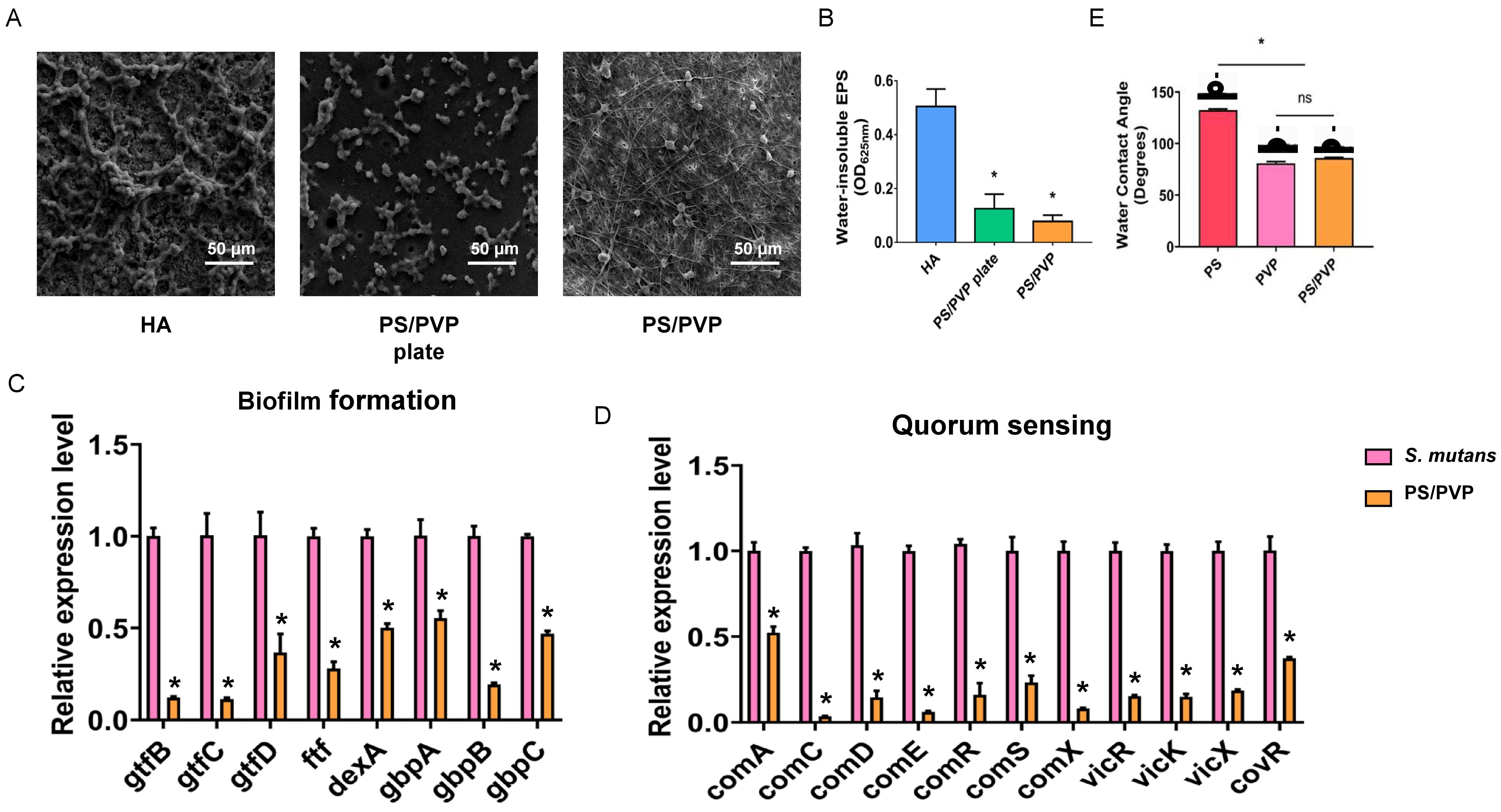

Supplement: rbae011_Supplementary_Data [file rbae011_supplementary_data.zip › Figure S2.tif]

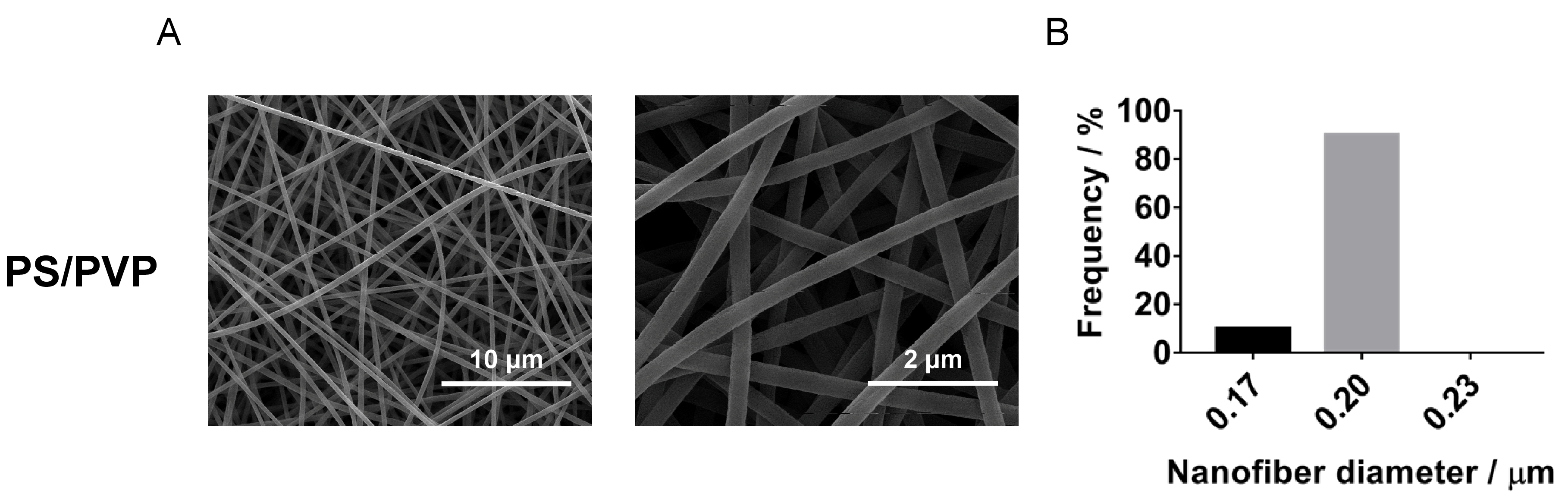

Supplement: rbae011_Supplementary_Data [file rbae011_supplementary_data.zip › Figure S1.tif]

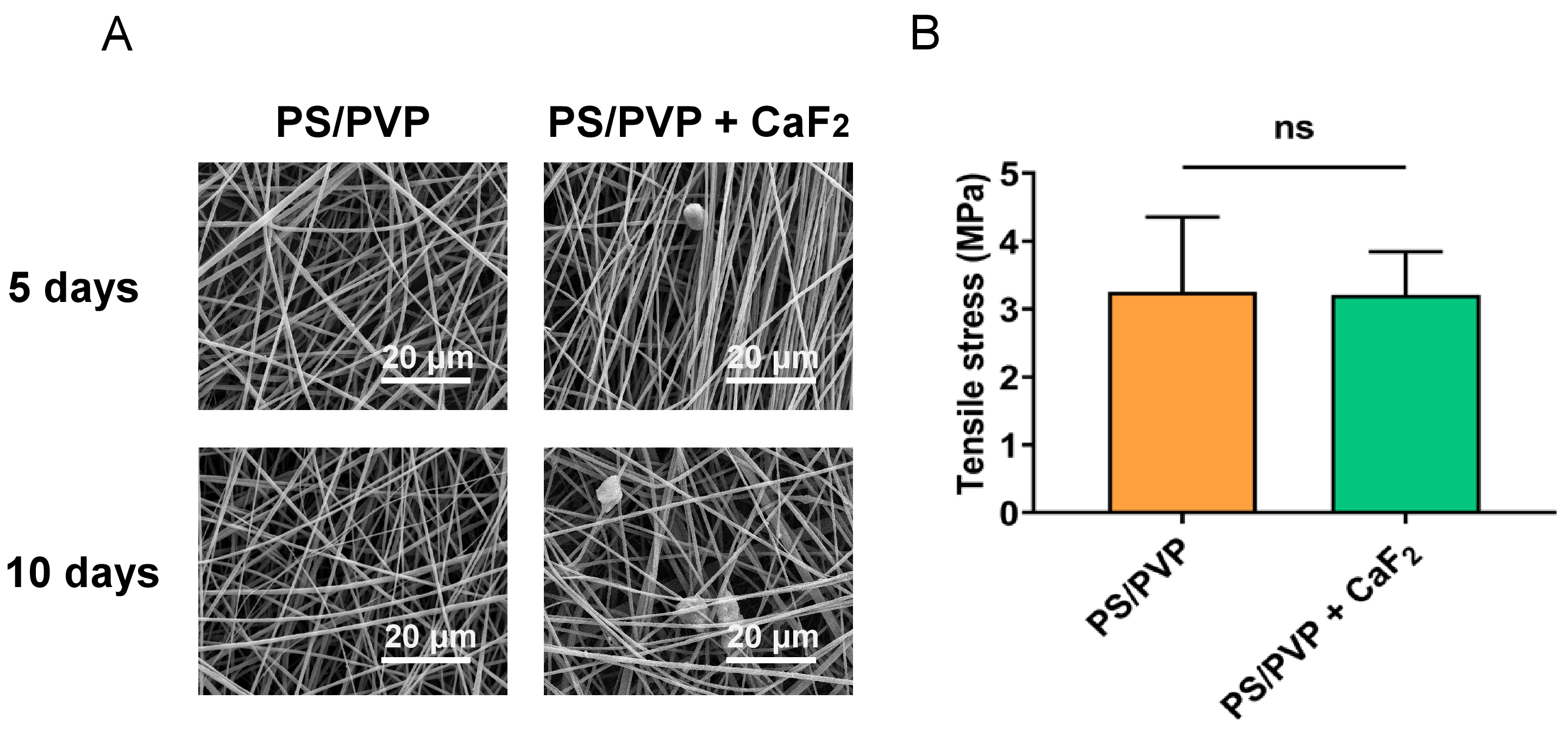

Supplement: rbae011_Supplementary_Data [file rbae011_supplementary_data.zip › Figure S3.tif]
